# Enhanced production of sucrose in the fast-growing cyanobacterium *Synechococcus elongatus* UTEX 2973

**DOI:** 10.1038/s41598-019-57319-5

**Published:** 2020-01-15

**Authors:** Po-Cheng Lin, Fuzhong Zhang, Himadri B. Pakrasi

**Affiliations:** 1 Department of Energy, Environmental & Chemical Engineering, Washington University, St. Louis, MO 63130 USA; 2 Department of Biology, Washington University, St. Louis, MO 63130 USA

**Keywords:** Applied microbiology, Bacterial genetics

## Abstract

Cyanobacteria are attractive microbial hosts for production of chemicals using light and CO_2_. However, their low productivity of chemicals is a major challenge for commercial applications. This is mostly due to their relatively slow growth rate and carbon partitioning toward biomass rather than products. Many cyanobacterial strains synthesize sucrose as an osmoprotectant to cope with salt stress environments. In this study, we harnessed the photosynthetic machinery of the fast-growing cyanobacterium *Synechococcus elongatus* UTEX 2973 to produce sucrose under salt stress conditions and investigated if the high efficiency of photosynthesis can enhance the productivity of sucrose. By expressing the sucrose transporter CscB, *Synechococcus* 2973 produced 8 g L^−1^ of sucrose with a highest productivity of 1.9 g L^−1^ day^−1^ under salt stress conditions. The salt stress activated the sucrose biosynthetic pathway mostly via upregulating the *sps* gene, which encodes the rate-limiting sucrose-phosphate synthase enzyme. To alleviate the demand on high concentrations of salt for sucrose production, we further overexpressed the sucrose synthesis genes in *Synechococcus* 2973. The engineered strain produced sucrose with a productivity of 1.1 g L^−1^ day^−1^ without the need of salt induction. The engineered *Synechococcus* 2973 in this study demonstrated the highest productivity of sucrose in cyanobacteria.

## Introduction

Microbial production of fuels and commodity chemicals provides alternative solutions to reduce the reliance on fossil fuel. However, the requirement of sugar feedstock is one of the challenges for sustainable bioproduction. Cyanobacteria are photosynthetic prokaryotes that use light, CO_2_, and trace amounts of minerals for growth. Compared to terrestrial plants, cyanobacteria have higher efficiencies to utilize solar energy^1^. In recent years, many synthetic biology tools have been developed for cyanobacteria^2^. These tools have enabled metabolic engineering of cyanobacteria to produce various chemicals, including fuels^3^, petrochemicals^4^, sugars^5^, fragrances^6^, and biopolymers^7^. Although cyanobacteria demonstrate the potential of converting CO_2_ into desired products, most of the reported titers and productivities are still too low for commercial applications^8,9^. A more efficient photosynthetic chassis is needed to improve CO_2_ utilization and carbon partitioning toward products.

**Figure 1:**
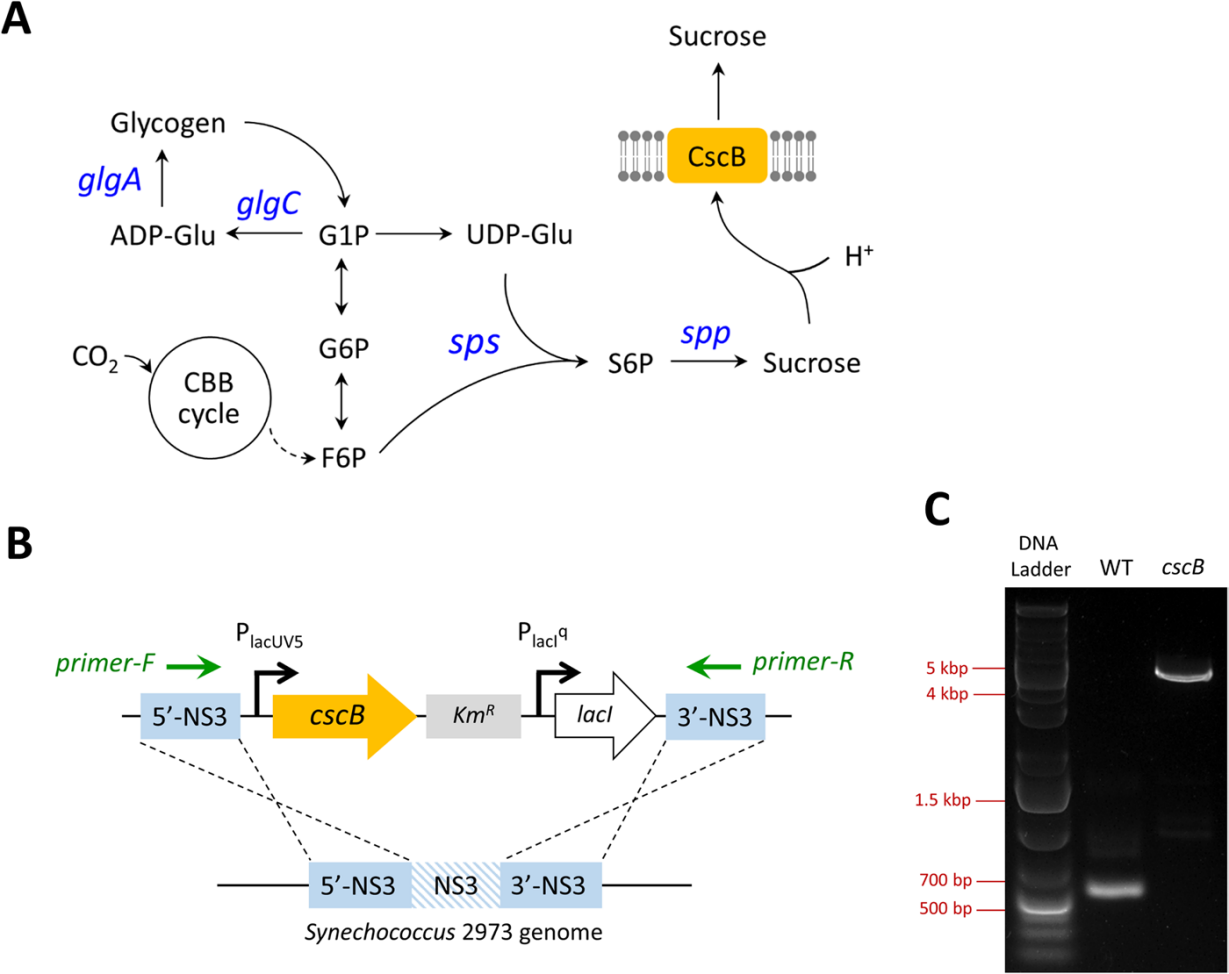
Engineering of *Synechococcus elongatus* UTEX 2973 for production of sucrose. (**A**) Metabolic pathways of carbohydrate biosynthesis in *Synechococcus* 2973. The sucrose permease CscB was expressed for sucrose excretion. Genes highlighted in blue encode enzymes for glycogen and sucrose production. In *Synechococcus* 2973, the SPS enzyme has both sucrose-phosphate synthase and sucrose-phosphate phosphatase activities. (**B**) Introduction of the *cscB* gene into the neutral site 3 (NS3). The primers used for PCR genotyping are highlighted in green. (**C**) PCR genotyping to confirm complete segregation of *cscB*. The wild type (WT) *Synechococcus* 2973 was used as a control. The PCR product of the WT gene is 514 base pairs (bp). No WT copy was present in the *cscB*-expressing strain. ADP-Glu, adenosine diphosphate glucose; CBB cycle, Calvin-Benson-Bassham cycle; CscB, sucrose permease; F6P, fructose 6-phosphate; G1P, glucose 1-phosphate; G6P, glucose 6-phosphate; S6P, sucrose 6-phosphate; UDP-Glu, uridine diphosphate glucose; *glgA*, glycogen synthase; *glgC*, ADP-glucose pyrophosphorylase; *spp*, sucrose-phosphate phosphatase; *sps*, sucrose-phosphate synthase.

Sucrose is an important feedstock in food industry and bioethanol production. Cyanobacteria synthesize sucrose as a compatible solute to tolerate high salt environments. By synthesizing sucrose, the osmotic pressure can be maintained to avoid desiccation in salt stress conditions. Studies of various cyanobacterial strains showed that more than 60 strains accumulate sucrose under high salt conditions^10^. In cyanobacterial cells, sucrose is synthesized from uridine diphosphate glucose (UDP-Glu) and fructose 6-phosphate (F6P) by sucrose-phosphate synthase (SPS) and sucrose-phosphate phosphatase (SPP) (Fig. 1A).

CscB is a sucrose/H^+^ symporter which belongs to the oligosaccharide: H^+^ symporter family of the Major Facilitator Superfamily^11^. Bacteria with CscB are able to take up sucrose by utilizing the H^+^ gradient across plasma membranes^12^. Ducat *et al*. reported the first study of using the sucrose permease CscB as a sucrose exporter in cyanobacteria^13^. Upon expression of the *E. coli* CscB in *Synechococcus elongatus* PCC 7942, the higher pH value in the extracellular medium creates a reversed proton gradient, which enables the engineered strain to export sucrose^13^. The *cscB*-expressing *Synechococcus* 7942 exported up to 2.6 g L^−1^ of sucrose with a rate of 0.9 g L^−1^ day^−1^ in high salt medium^13^.

Additionally, a sucrose biosynthetic pathway was also engineered in *Synechocystis* sp. PCC 6803 by overexpressing the *sps* and *spp* genes, leading to a 2-fold enhancement in intracellular sucrose concentration^14^. Importantly, this study identified that the SPS-catalyzed reaction is the rate-limiting step of sucrose biosynthesis in cyanobacteria^14^. Recently, the fast-growing cyanobacterium *Synechococcus elongatus* UTEX 2973 was engineered for sucrose production by expressing the *cscB* gene^5^. However, the fast-growing property of *Synechococcus* 2973 did not lead to a higher sucrose titer or productivity compared to those from its closely-related strain *Synechococcus* 7942^5^. Compared to *Synechococcus* 7942, *Synechococcus* 2973 has a higher carbon flux in the sugar phosphate pathway^15^ and accumulates a greater amount of glycogen^5,16^. Hence, sucrose productivity in *Synechococcus* 2973 is expected to be higher than that in *Synechococcus* 7942. Therefore, these results demanded further research to uncover the potential of sugar production in this fast-growing strain.

Inspired by these facts, we explored the capability of sucrose production in engineered *Synechococcus* 2973 and investigated whether the rapid growth rate and strong carbon fluxes in sugar metabolism can lead to a higher production titer of sucrose. In this work, we reported a high sucrose productivity to 1.9 g L^−1^ day^−1^ by the engineered *Synechococcus* 2973. Moreover, we discussed the relationship between glycogen synthesis and sucrose production, providing insights on carbon partitioning of cyanobacteria in salt stress conditions. Finally, we further engineered *Synechococcus* 2973 to produce sucrose without induction by salt stress. We determined that SPS and SPP are both important enzymes in sucrose biosynthesis, and the expression level of SPP is critical for enhanced sucrose production.

## Results

### Engineering of *Synechococcus* 2973 for sucrose production

**Figure 2:**
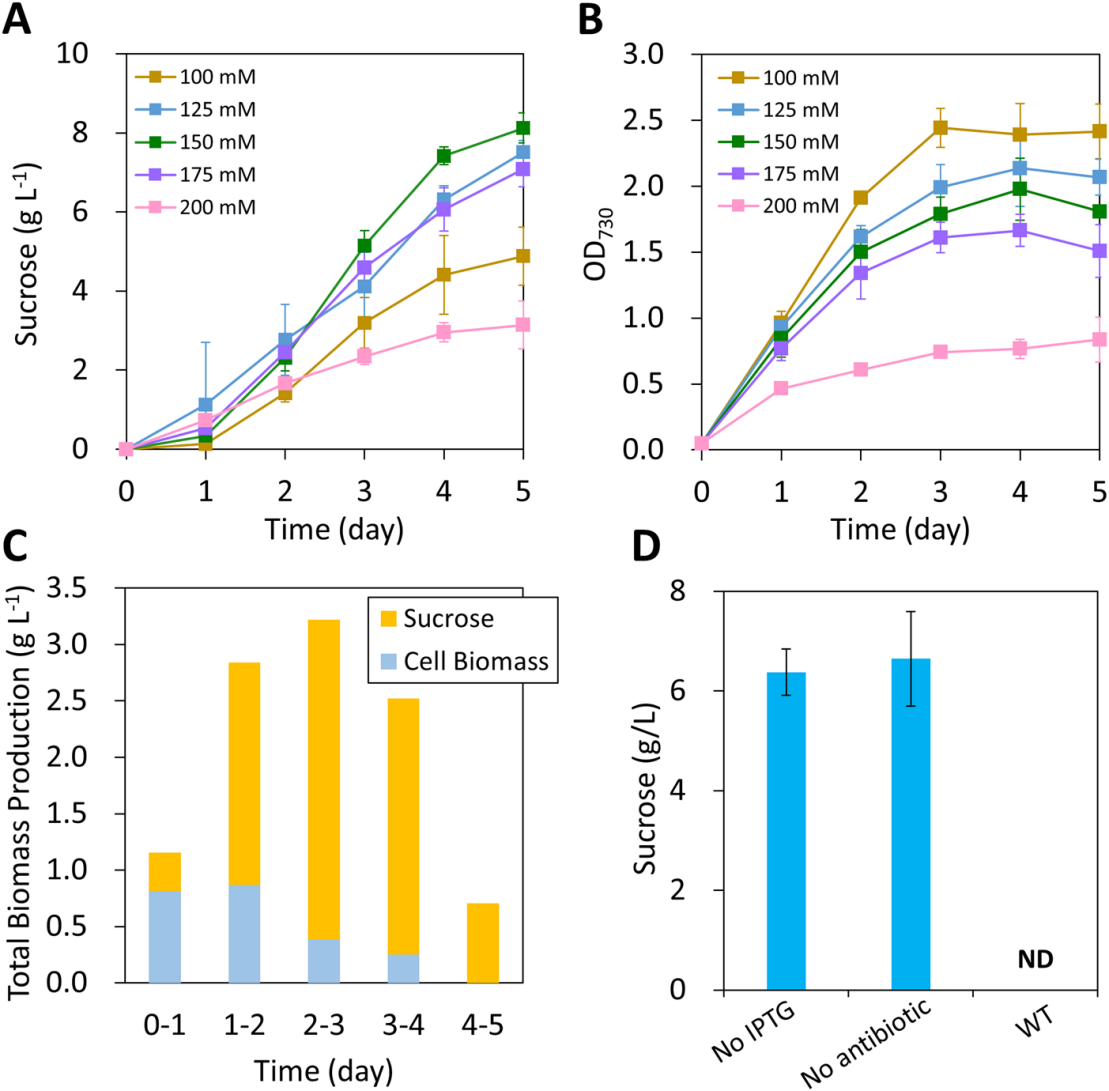
Sucrose production in *cscB*-expressing *Synechococcus* 2973. (**A**) Time-course of sucrose production in the *cscB*-expressing strain in BG11 medium with various concentrations of NaCl. (**B**) OD_730_ of the *cscB*-expressing strain. (**C**) Carbon partitioning between sucrose and cell biomass (BG11 with 150 mM NaCl). (**D**) Sucrose production in different culture conditions with 150 mM NaCl (5 days). The WT *Synechococcus* 2973 was included as a control. The data represents mean ± sd from three biological replicates. ND, not detected.

To engineer a sucrose-exporting *Synechococcus* 2973 strain, the sucrose permease *cscB* gene was cloned into a neutral site 3 (NS3)-targeting plasmid^17^, which was then introduced into *Synechococcus* 2973 via bacterial conjugation (Fig. 1B). A fully segregated strain was obtained by re-patching the plates multiple times, and the corrected genome modification was confirmed by PCR genotyping (Fig. 1C). To test sucrose production in *Synechococcus* 2973, the engineered 2973-*cscB* strain (Table 1) was grown in BG11 with 100–200 mM NaCl (Fig. 2A). IPTG (1 mM) was added to induce *cscB* gene expression. The supernatants were collected for sucrose analysis. The highest sucrose titer, 8 g L^−1^ in 5 days, was observed in BG11 with 150 mM NaCl. The highest volumetric productivity was 1.9 g L^−1^ day^−1^ in 4 days, which corresponds to a sucrose yield of 3.1 g.g^−1^ cell biomass. This productivity is over 2-fold higher than that from the closely-related strain *Synechococcus* 7942 (Supplementary Fig. S1). The pH of the medium was lower under sucrose-producing conditions because protons are exported with sucrose by the CscB sucrose/H^+^ symporter (Supplementary Table S1). Additionally, we observed that salt stress resulted in impaired growth of *Synechococcus* 2973 (Fig. 2B), leading to decreased OD_730_ values as NaCl concentrations increased.

**Table 1:** Plasmids and strains used in this study.

Name	Description	Reference
**Plasmids**		
pRL443	conjugal plasmid for bacterial conjugation	^29^
pRL623	helper plasmid for bacterial conjugation	^29^
pSL2985	P_lacUV5_-*cscB* (*E. coli*), *lacI*, NS3-targeting [Cm^R^]	^13^
pSL3005	P_lacUV5_-*cscB* (*E. coli*), *lacI*, NS3-targeting [Kan^R^]	This study
pSL3037	P_lacUV5_-*cscB* (*E. coli*), *lacI*, bom site, NS3-targeting [Kan^R^]	This study
pSL3360	P_trc1O_-*sps* (*Synechocystis* 6803), RSF1010 plasmid [Gm^R^]	This study
pSL3361	P_trc1O_-*sps*-*spp* (*Synechocystis* 6803), RSF1010 plasmid [Gm^R^]	This study
***Synechococcus*** **2973 strains**		
2973-*cscB*	NS3::P_lacUV5_*-cscB* (created using pSL3037)	This study
2973-*cscB*-*spp*	2973-*cscB* strain with plasmid pSL3360	This study
2973-*cscB*-*sps*-*spp*	2973-*cscB* strain with plasmid pSL3361	This study

To investigate how much photosynthetically fixed carbon is directed to sucrose production under salt stress conditions, we analyzed carbon partitioning between sucrose and cell biomass (Fig. 2C). During the first 24 hours of growth, 71% of the fixed carbon (0.8 g L^−1^) was used for cell biomass synthesis, and only 0.3 g L^−1^ of sucrose was produced in this period. From day 1 to day 4, the sucrose titer increased dramatically. During each of these days, the sucrose productivity was greater than 2 g L^−1^ day^−1^. The highest amount of sucrose was produced between day 3–4, during which sucrose productivity reached 2.8 g L^−1^. After day 3, over 88% of the fixed carbon was directed to sucrose. The titer decreased during day 4–5, in which only 0.7 g L^−1^ sucrose was produced.

Furthermore, we tested the capacity of sucrose production without the addition of IPTG or the antibiotic kanamycin. Without IPTG induction, the 2973-*cscB* strain produced 6.4 g L^−1^ sucrose (Fig. 2D), which is 21% lower than that under IPTG-induced conditions (Fig. 2A). A recent study reported that IPTG inducible promoters (*trc*, *lac*, and *LlacO-1*) are leaky in *Synechococcus* 2973^18^. Our data also indicate that the *lacUV5* promoter is leaky in *Synechococcus* 2973. Similarly, the 2973-*cscB* strain produced 6.6 g L^−1^ sucrose without addition of kanamycin (Fig. 2D). Although the *cscB* gene was inserted into the genome, this result show that kanamycin is required to maintain high sucrose productivity in *Synechococcus* 2973.

### Investigation of carbohydrate metabolism in sucrose-producing *Synechococcus* 2973

**Figure 3:**
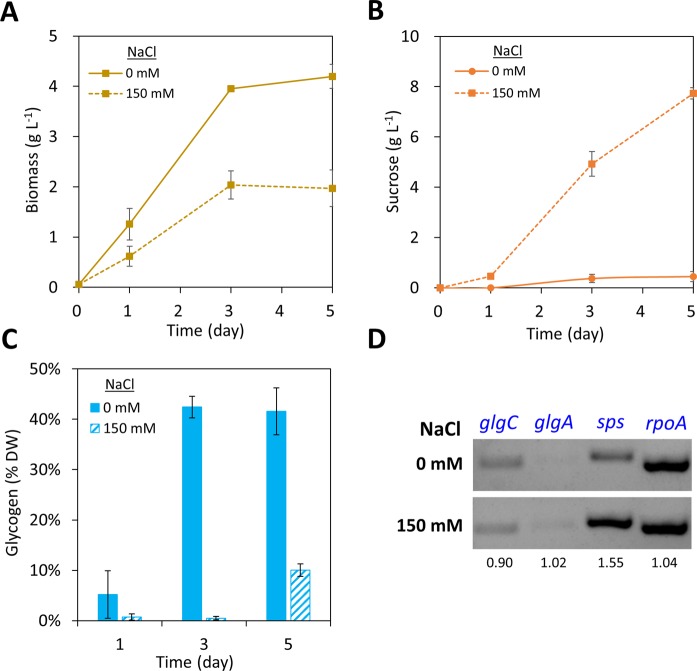
Analysis of carbohydrate metabolism in *Synechococcus* 2973 *cscB*-expressing strain. Cells were grown in BG11 medium with or without addition of 150 mM NaCl. (**A**) Biomass accumulation. (**B**) Sucrose production. (**C**) Glycogen content. (**D**) Semi-quantitative RT-PCR for monitoring gene expression in glycogen and sucrose syntheses. The housekeeping gene *rpoA* was used as a control. The numbers below the gel represent the relative band intensities from samples growing with 150 mM NaCl versus 0 mM NaCl. The full-length gels are presented in Supplementary Fig. S2. The data in Panels A–C represents mean ± sd from three biological replicates. DW, cell dry weight; *rpoA*, RNA polymerase subunit α.

After its exponential growth phase, *Synechococcus* 2973 accumulates a significant amount of glycogen to reserve excess fixed carbon^16^. To further understand the carbon allocation between glycogen and sucrose, we analyzed the glycogen content in the sucrose-producing *Synechococcus* 2973 strain. The 2973-*cscB* cells were grown in BG11 with or without 150 mM NaCl, and the amounts of glycogen and sucrose were analyzed. The cell biomass was 2-fold higher in BG11 medium without addition of NaCl (Fig. 3A). In the absence of NaCl, *Synechococcus* 2973 produced only 0.4 g L^−1^ of sucrose in 5 days, which is 19-fold lower than that in the presence of the salt (Fig. 3B). In contrast, the glycogen content significantly decreased under sucrose-producing conditions. In the presence of 150 mM NaCl, the 2973-*cscB* strain accumulated glycogen less than 1% of cell dry weight (DW) during day 1 and day 3 (Fig. 3C). On day 5, the glycogen content increased to 10% of DW, corresponding to 0.2 g L^−1^ of glycogen (40-fold lower than sucrose). Without addition of NaCl, cells accumulated glycogen to more than 40% of DW after 3 days (Fig. 3C). These observations further confirmed that salt stress redirected carbon fluxes from glycogen accumulation to sucrose production in this engineered strain.

We next conducted a semi-quantitative RT-PCR to examine transcription of genes involved in glycogen and sucrose biosynthesis, including *glgC*, *glgA*, and *sps* (Fig. 1A). GlgC (ADP-glucose pyrophosphorylase) and GlgA (glycogen synthase) are the enzymes responsible for glycogen biosynthesis. In *Synechococcus elongatus*, the SPS enzyme has both sucrose-phosphate synthase and sucrose-phosphate phosphatase activities^19^. Therefore, expression of the *sps* gene was monitored. The housekeeping gene *rpoA* (encoding RNA polymerase subunit α) was used as a control for this RT-PCR experiment^20^. Transcript levels of *glgC* and *glgA* were not significantly different with or without addition of NaCl (Fig. 3D). In contrast, the sucrose synthesis gene *sps* was highly expressed under salt stress condition (Fig. 3D), suggesting that increased sucrose production in the salt medium is due to upregulation of the sucrose biosynthetic pathway.

### Engineering of the sucrose biosynthetic pathway

**Figure 4:**
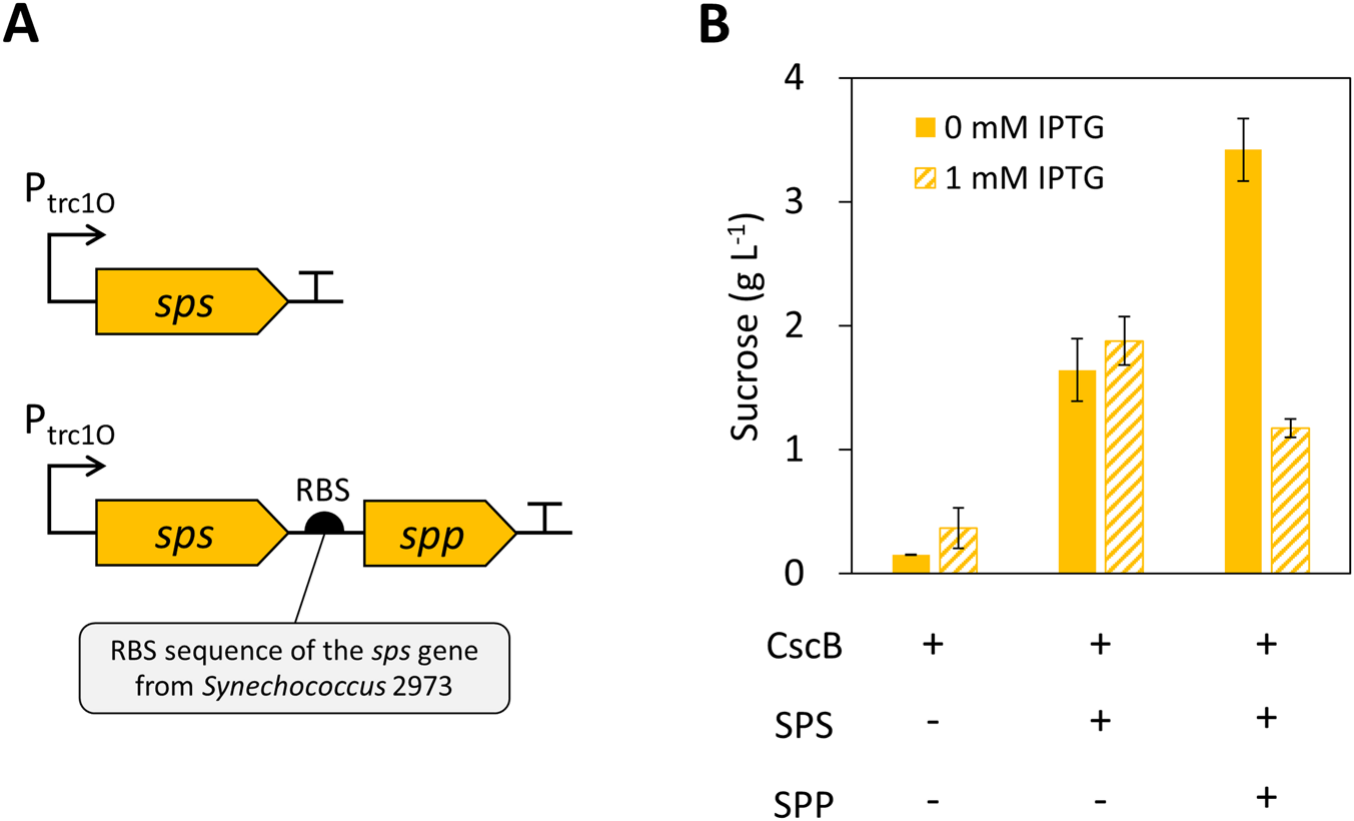
Engineering of the sucrose biosynthetic pathway in *Synechococcus* 2973. (**A**) Schematics of expressing the *Synechocystis* 6803 *sps* and *spp* genes in the 2973-*cscB* strain. (**B**) Sucrose production in *sps* and *sps*-*spp* expression strains. Cells were cultured in BG11 medium for 3 days without addition of NaCl. The “+” symbol indicates that the strain contains the indicated overexpressed enzyme(s). The data represents mean ± sd of three biological replicates. RBS, ribosome binding site.

In cyanobacteria, salt stress is required to induce sucrose production. The sucrose biosynthesis gene *sps* is up-regulated in salt BG11 medium (Fig. 3D). To expand the applications of sucrose production in cyanobacteria, we engineered *Synechococcus* 2973 to produce sucrose in BG11 medium without added NaCl. The sucrose biosynthetic pathway from *Synechocystis* sp. PCC 6803 was expressed in the 2973-*cscB* strain (Fig. 4A). It is known that overexpression of the *sps* and *spp* genes from *Synechocystis* 6803 leads to a 2-fold improvement in intracellular sucrose production under salt stress conditions^14^. To express the *Synechocystis* 6803 *sps* and *spp* genes in *Synechococcus* 2973, we cloned them into the self-replicating RSF1010 plasmid^21^ under the control of an IPTG-inducible *trc1O* promoter. The plasmids expressing either *sps* alone or *sps*-*spp* genes were then introduced into the 2973-*cscB* strain (Table 1). Both *sps* and *sps*-*spp* overexpression strains were able to produce sucrose in BG11 medium without additional NaCl (Fig. 4B). In the absence of IPTG, the *sps* and *sps*-*spp* strains produced 1.6 g L^−1^ and 3.4 g L^−1^ of sucrose in 3 days, respectively, which were 11-fold and 23-fold higher than the control 2973-*cscB* strain (Fig. 4B). For the *sps* strain, the sucrose titers had no significant difference with or without addition of 1 mM IPTG. Interestingly, the sucrose titer decreased significantly upon addition of 1 mM IPTG in the *sps*-*spp* strain (Fig. 4B), indicating that full induction of *sps* and *spp* expression may be detrimental to sucrose production.

## Discussion

During this study, we engineered the fast-growing cyanobacterium *Synechococcus* UTEX 2973 to produce sucrose. By overexpressing the sucrose permease CscB, the engineered strain produced over 8 g L^−1^ of sucrose with the highest productivity of 1.9 g L^−1^ day^−1^ in 4 days (Fig. 2A). Remarkably, a significant amount of sucrose was produced at the stationary growth phase. From day 2 to day 5, 5.8 g L^−1^ of sucrose was produced, while the cell biomass increased only 0.7 g L^−1^ (Fig. 2C). Nearly 90% of the photosynthetic carbon was directed to sucrose synthesis. Surprisingly, the self-shading of light in dense cell cultures had no significant impact on sucrose production. We reasoned that the high productivity of sucrose is a result of push-and-pull of carbon fluxes toward sucrose synthesis. In *Synechococcus* 2973, the high photosynthetic efficiency^16^ and strong sugar phosphates pathways^15^ provide a strong pushing force for sucrose production. Meanwhile, expression of the sucrose permease CscB generates a carbon sink by exporting sucrose out of the cells, thus resulting in continuous sucrose production to avoid desiccation in salt stress conditions.

Compared to all other chemicals produced from cyanobacteria, sucrose production in this study has the highest productivity. Moreover, this is the first study demonstrating cyanobacteria can produce a chemical with a rate over 1 g L^−1^ day^−1^ using light and CO_2_. A recent study reported 12.6 g L^−1^ of 2,3-butanediol production with a rate of 1.1 g L^−1^ day^−1^ from *Synechococcus* 7942^22^. However, the strain was cultured under photomixotrophic conditions with 15 g L^−1^ of glucose added as an additional carbon source. The sucrose production in *Synechococcus* 2973 in the current study used CO_2_ as the sole carbon source.

Previously engineered *Synechococcus* 2973^5^ produced sucrose at 0.9 g L^−1^ day^−1^, which is 2.1-fold lower than that from our 2973-*cscB* strain (Supplementary Fig. S1). The discrepancy between the two studies is presumably due to differences in strain design and experimental conditions. In their study, the *cscB* was controlled by an *E. coli* trp/lac promoter^17^, whereas the 2973-*cscB* strain in our study used the *lacUV5* promoter to express *cscB*^13^. Moreover, the inoculum preparation and experimental conditions are different. Their experiments were initiated by salt induction of late exponential phase cultures (cell biomass = 1.9 g L^−1^ dry weight)^5^. The cultures were grown in 3% CO_2_ and 250 μmol photons m^−2^ s^−1^ light in a column bioreactor^5^. In our study, the 2973-*cscB* strain was first acclimated in BG11 medium with 150 mM NaCl for 24 hours, and the experiments were initiated at an OD_730_ of 0.05 (equivalent to 0.06 g L^−1^ dry weight) with IPTG induction. The 2973-*cscB* strain was cultured in 0.5–0.6% CO_2_ and 250 μmol photons m^−2^ s^−1^ light in shaker flasks. We reasoned that the acclimation of cells to salt stress medium enabled the *cscB*-expressing strain to produce a higher amount of sucrose. Since the salt stress inhibits the activity of photosystems^23^ and impairs the growth of *Synechococcus* 2973 (Fig. 2B), the previously reported salt induction strategy may not be able to unlock the potential of sucrose production in *Synechococcus* 2973. The promoter choice for *cscB* expression and salt acclimation strategy in our study have led to a higher sucrose productivity by *Synechococcus* 2973.

The levels of glycogen and sucrose are opposite when the 2973-*cscB* strain were grown with or without salt stress (Fig. 3B,C). This suggests that glycogen and sucrose syntheses compete for carbon substrates, and the competition can be affected by culture media conditions. The semi-quantitative RT-PCR results showed that the *sps* gene was significantly upregulated by salt induction (Fig. 3D), which led to significant increase in sucrose production (Fig. 3B). However, expression of the glycogen synthesis genes (*glgC* and *glgA*) remained similar upon salt induction (Fig. 3D), indicating that the decreased glycogen content was not due to downregulation of glycogen synthesis. This also suggests that the sucrose biosynthetic pathway is more efficient than the glycogen synthesis pathway in utilizing the carbon substrates in the 2973-*cscB* strain. In fact, our results corroborate the finding that deletion of the glycogen synthesis pathway has led to a limited improvement on sucrose production in cyanobacteria^13^. Since a negligible amount of glycogen is produced under salt stress conditions (Fig. 3C), the sucrose titer is unlikely to be further improved by deletion of the glycogen pathway. In fact, a recent study reported that glycogen synthesis may serve as a carbon pool for sucrose production in cyanobacteria^24^. By using a theophylline-induced riboswitch system, reduced expression of *glgC* led to decreased sucrose titer in *Synechococcus* 7942^24^.

By overexpressing the sucrose biosynthesis genes, sucrose production of 2973-*cscB* cells increased significantly in BG11 medium (Fig. 4B). This is the first study to engineer cyanobacteria for sucrose production without induction of salt stress. The sucrose-producing cyanobacteria can be used as carbon feedstock for heterotrophs to produce useful chemicals^25^. However, the salt stress media may not be compatible with the culture media for heterotrophs. Engineering a sucrose-producing strain without the need of salt induction could expand the application of using cyanobacteria for co-culture systems. Expression of *sps* and *spp* improved the sucrose titer by 23-fold with a productivity of 1.1 g L^−1^ day^−1^ (Fig. 4B). With or without addition of 1 mM IPTG, the *sps* strain produced similar amounts of sucrose (1.6–1.8 g L^−1^). Notably, sucrose production of the *sps*-*spp* coexpression strains were 1.2 g L^−1^ and 3.4 g L^−1^ with or without addition of IPTG, respectively (Fig. 4B). The productivity increased 2.9-fold in the absence of IPTG, suggesting that the expression level of *spp* greatly affects the sucrose titer. The *cscB* and *sps-spp* genes were both controlled by IPTG-inducible promoters, while only one copy of the *lacI* repressor gene was expressed in the strain (Figs. 1B and 4A). The amount of LacI repressor may be insufficient to control gene expression of all 3 genes. Hence, leaky expression of the genes (*cscB*, *sps* and *spp*) led to lower gene expression but significantly improved sucrose production when compared to full induction of the *spp* gene in the present of 1 mM IPTG (Fig. 4B). We also observed impaired growth of the *sps*-*spp* strain with 1 mM IPTG induction (Supplementary Table S2), suggesting that overexpression of the *spp* gene may cause cell toxicity and reduce sucrose productivity. It is known that SPS is the rate-limiting enzyme of sucrose biosynthesis in cyanobacteria^14^. Our results suggest that SPP also plays an important role in sucrose production, in that its expression level is critical for enhancing sucrose production.

## Conclusions

We have demonstrated the significant potential of engineered *Synechococcus* UTEX 2973 for photosynthetic sugar production. Expression of the sucrose transporter CscB led to continuous sucrose production with a rate of 1.9 g L^−1^ day^−1^ in salt stress conditions. Similar concepts can be applied to harness the strong sugars fluxes in *Synechococcus* 2973 to produce other valuable sugar compounds^26^. The salt stress activates the sucrose biosynthetic pathway, which redirects a significant portion of the fixed CO_2_ toward sucrose production rather than biomass and glycogen accumulation. By engineering the sucrose synthesis pathway in *Synechococcus* 2973, the mutant strains produced sucrose without salt stimulation, which could expand the versatility of using cyanobacteria for sugar production. Furthermore, we identified SPS and SPP as important enzymes for sucrose synthesis in *Synechococcus* 2973, while the expression level of SPP is particularly critical for enhancing sucrose production.

## Methods

### Growth conditions

*Synechococcus elongatus* UTEX 2973 was grown in BG11 liquid medium at 38 °C, 250 μmol photons m^−2^ s^−1^ light, and 0.5–0.6% CO_2_ in an AlgaeTron growth chamber (Photon Systems Instruments, Czech Republic). For the mutant strains, appropriate antibiotics (10 μg/mL kanamycin and 4 μg/mL gentamicin) were applied in BG11 agar plates or liquid medium. To test sucrose production, strains were first grown in BG11 medium for 24 hours, and then diluted 40-fold to BG11 with 150 mM NaCl to grow for another 24 hours. Afterwards, the salt-acclimated cultures were diluted to an OD_730_ of 0.05 (0.06 g L^−1^ cell dry weight) in 10 mL of BG11 medium in 50-mL flasks with desired concentrations of NaCl to initiate the experiments. Water was added daily to compensate for evaporation of BG11 medium. The evaporation rate is 5–7% per day.

### Strain construction

All cloning was conducted using the Gibson isothermal DNA assembly method^27^. The sucrose permease *cscB* gene from *E. coli* (ATCC 700927) was introduced into the neutral site 3 (NS3) of *Synechococcus* UTEX 2973^17^. The *cscB* was controlled by the *lacUV5* promoter. A lac repressor gene (*lacI*) was included in the plasmid to control *cscB* expression. The *cscB* NS3-targeting plasmid was a.pngt from Prof. Daniel Ducat at Michigan State University^13^. The kanamycin resistance cassette was cloned into the plasmid to replace the chloramphenicol resistance gene. Further, the basis of mobility (bom) sequence derived from plasmid pBR322 was cloned into the plasmid. The bom site is required for gene transfer via bacterial conjugation^28^. The workflow of plasmid construction is described in Supplementary Fig. S3. The sucrose biosynthesis pathway was expressed in the RSF1010 self-replicating plasmid^21^. The *sps* (*sll0045*) and *spp* (*slr0953*) genes were amplified from the genomic DNA of *Synechocystis* 6803. The ribosome binding site (RBS) sequence of the *sps* gene in *Synechococcus* 2973 (20 base pairs proceeding the coding sequence) was used to control translation of *spp* (Fig. 4A). The *trc1O* promoter was used to control expression of the genes. The plasmids and mutant strains used in this study are listed in Table 1.

Bacterial conjugation was used to introduce the *cscB* NS3-targeting or self-replicating plasmids (Table 1) into *Synechococcus* 2973. *E. coli* HB101 with plasmid pRL443 was the conjugal strain, whereas *E. coli* HB101 with plasmid pRL623 and the plasmid carrying the genes of interest was the helper strain. Both *E. coli* strains and *Synechococcus* 2973 were grown in LB and BG11 liquid cultures overnight, respectively. 100 μL of *E. coli* cultures and 400 μl of *Synechococcus* 2973 culture (OD_730_ = 0.5) were washed twice with sterilized water and BG11 medium, respectively. Centrifugation was performed at 4,000 × *g* for 5 mins. The *E. coli* cells were gently stirred during the wash step. The washed *E. coli* and *Synechococcus* 2973 strains were mixed together with 200 μL of BG11 medium and transferred onto a nitrocellulose membrane (0.45 μm pore size, Millipore Sigma, USA) on a BG11 + 5% LB (v/v) agar plate. The plates were incubated at 38 °C, 100 μmol photons m^−2^ s^−1^ light, and 0.5% CO_2_ overnight. The membrane was transferred to new BG11 agar plates with appropriate antibiotics (50 μg/mL kanamycin and 4 μg/mL gentamicin). Colonies appeared on the membranes after 3–5 days of incubation.

### Semi-quantitative RT-PCR

RNA was extracted from the 2973-*cscB* cultures using RNAwiz kit (Ambion/Thermo Fisher Scientific). The RNA samples were treated with DNase. Afterwards, cDNA was synthesized using a Superscript II Reverse Transcriptase kit (Thermo Fischer Scientific). PCR was performed at 24 cycles for semi-quantitative analysis of mRNA transcripts. The band intensities were quantified using ImageJ software. The primers used for PCR are listed in Supplementary Table S3.

### Measurements of sucrose, glycogen and biomass

Sucrose in the supernatant was measured using the sucrose/D-glucose assay kit (Megazyme). The glycogen assay was based on the method described in our previous study^16^. The correlation of OD_730_ with cell dry weight was DW (g L^−1^) = 1.3244 × OD_730_–0.2459 (Supplementary Fig. S4).

## Supplementary information

Supplementary Information.
