# Supplementary material for: Enhanced production of sucrose in the fast-growing cyanobacterium Synechococcus elongatus UTEX 2973
Source: Sci Rep. 2020 Jan 15;10:390. doi: 10.1038/s41598-019-57319-5 (PMC6962321; doi:10.1038/s41598-019-57319-5)
Supplement: Supplementary file 1 — Supplementary Information. [file 41598_2019_57319_MOESM1_ESM.pdf]

Supplementary Information for:

Enhanced production of sucrose in the fast-growing cyanobacterium *Synechococcus elongatus* UTEX 2973

Po-Cheng Lin[1], Fuzhong Zhang[1] and Himadri B. Pakrasi[2*]

[1]Department of Energy, Environmental & Chemical Engineering; [2]Department of Biology, Washington University, St. Louis, MO 63130, USA

Correspondence should be addressed to H.B.P (email:pakrasi@wustl.edu)

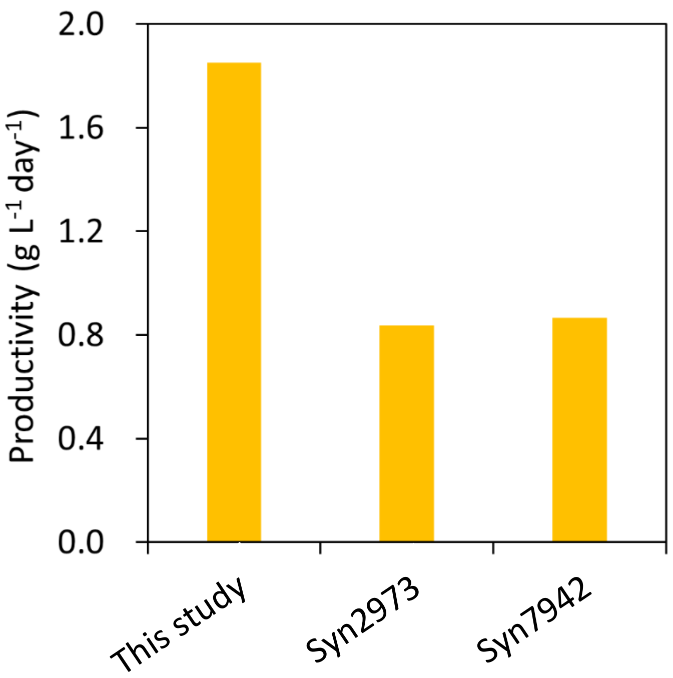

Figure S1. Comparison of sucrose productivity in cyanobacterial strains. Syn2973, *Synechococcus* 2973[1]; Syn7942, *Synechococcus* 7942[2].

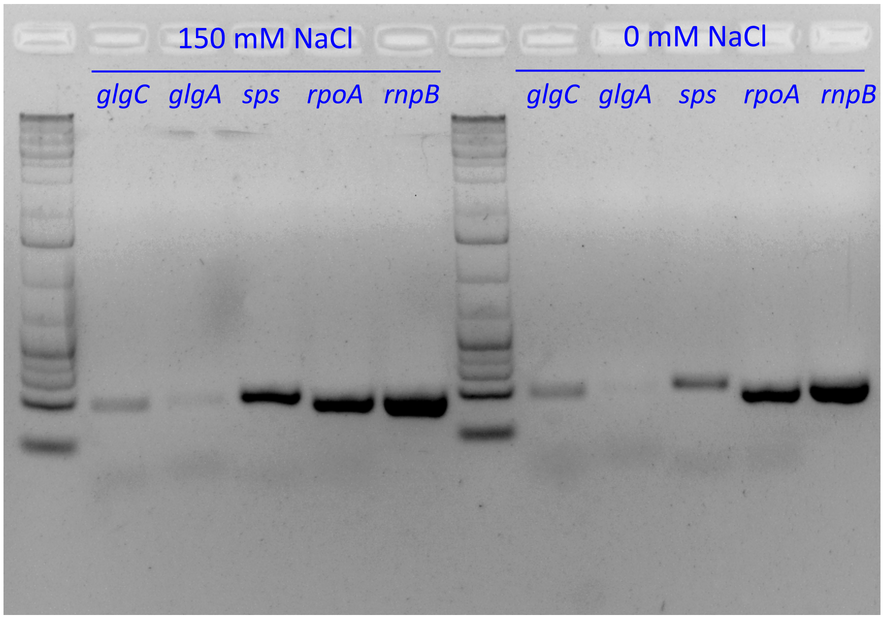

Figure S2. Agarose gel of semi-quantitative RT-PCR results for monitoring gene expression in glycogen and sucrose syntheses. The *Synechococcus* 2973 *cscB*-expressing strain was grown in BG11 medium with or without 150 mM NaCl for 70 hours. The housekeeping genes *rpoA* and *rnpB* were used as controls. A 1 Kb Plus DNA Ladder (ThermoFisher) was used.

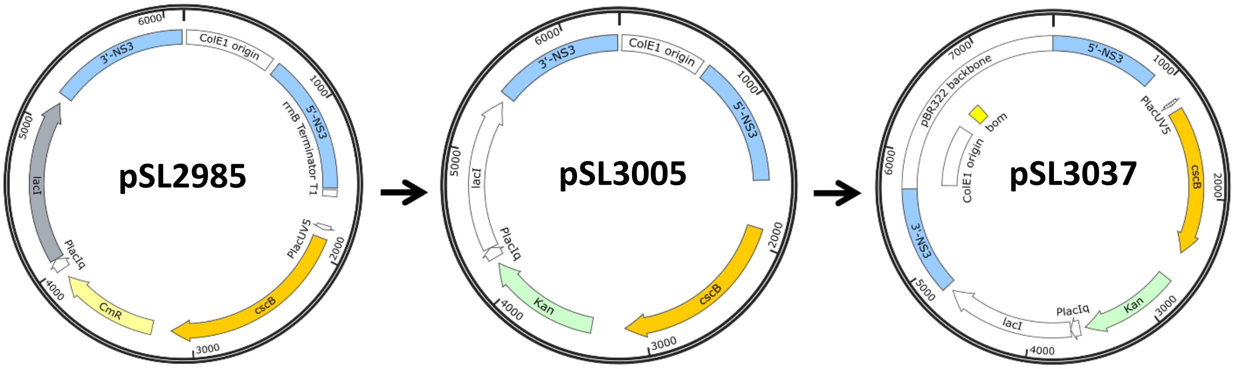

Figure S3. Workflow of plasmid construction.

Plasmid pSL2985

- This plasmid is from Prof. Daniel Ducat[2].
- The Cm^R cassette was replaced because the helper plasmid pRL623 for bacterial conjugation also uses Cm^R cassette.

Plasmid pSL3005

- The Km^R cassette was cloned into the plasmid to replace the Cm^R cassette.

Plasmid pSL3037

- The basis of mobility (bom) sequence derived from plasmid pBR322 was cloned into the plasmid.
- This plasmid was introduced into *Synechococcus* 2973 via bacterial conjugation.

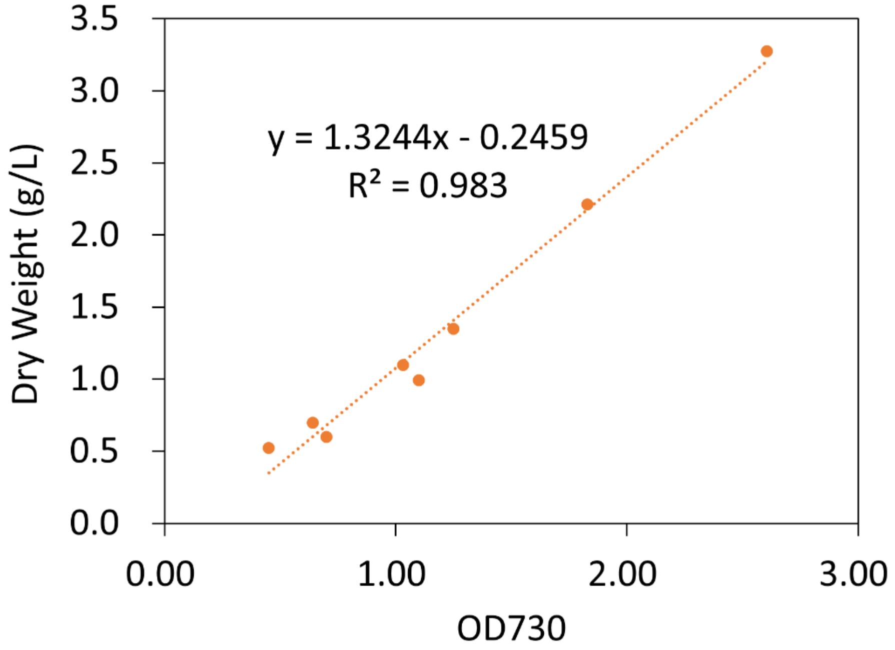

Figure S4. Correlation of cell dry weight (DW) with OD_{730} for *Synechococcus* 2973 *cscB*-expressing strain.

Table S1. The pH values of *Synechococcus* 2973 *cscB*-expressing cultures with or without 150 mM NaCl

Time (day)	150 mM NaCl	0 mM NaCl
1	8.20	8.55
3	8.55	8.75
5	8.65	8.80

Table S2. OD_{730} of *Synechococcus* 2973 sucrose-producing strains growing in BG11 medium for 3 days

Strains	0 mM IPTG	1 mM IPTG
2973-*cscB*	3.3	3.2
2973-*cscB-sps*	2.8	0.8
2973-*cscB-sps-spp*	2.6	0.6

Table S3. Primers for semi-quantitative RT-PCR

Primer name	Sequence (5’ - 3’)
glgC-F	GGCCTGATGAAGCTAGATGG
glgC-R	CCCGCTTGAAGACGTAGATG
glgA-F	GGTTGGTCGATACGGTCTTC
glgA-R	CTCAGATCAACGGCCATACC
sps-F	CAGGAAGTGGAGGAGCAATAC
sps-R	CGACAGAGGCAGAGAATTTGAG
rpoA-F	GGCTGCTGATGTTGACTTTG
rpoA-R	AAGTCTAGGGCAGTCGTTTC
rnpB-F	GGTCCATAAACGGAACAGGTAAA
rnpB-R	GGAGAGTGCCACAGAAACATAC

References

1 Song, K., Tan, X., Liang, Y. & Lu, X. The potential of *Synechococcus elongatus* UTEX 2973 for sugar feedstock production. *Applied microbiology and biotechnology* **100**, 7865-7875, doi:10.1007/s00253-016-7510-z (2016).

2 Ducat, D. C., Avelar-Rivas, J. A., Way, J. C. & Silver, P. A. Rerouting carbon flux to enhance photosynthetic productivity. *Applied and environmental microbiology* **78**, 2660-2668, doi:10.1128/AEM.07901-11 (2012).
